# The future of Japanese encephalitis vaccination: expert recommendations for achieving and maintaining optimal JE control

**DOI:** 10.1038/s41541-021-00338-z

**Published:** 2021-06-15

**Authors:** Kirsten S. Vannice, Susan L. Hills, Lauren M. Schwartz, Alan D. Barrett, James Heffelfinger, Joachim Hombach, G. William Letson, Tom Solomon, Anthony A. Marfin, the Japanese encephalitis vaccination experts panel, Katie Anderson, Marc Fischer, Kim Fox, Julie Jacobson, Jayantha Liyanage, Florian Marks, Ike Ogbuanu, Piyanit Tharmaphornpilas

**Affiliations:** 1 Consultant, PATH, Seattle, WA USA; 2 Arboviral Diseases Branch, Centers for Disease Control and Prevention, Fort Collins, CO USA; 3 Schwartz Consulting, Seattle, WA USA; 4 Sealy Institute for Vaccine Sciences, Department of Microbiology & Immunology, University of Texas Medical Branch, Galveston, TX USA; 5 WHO Regional Office for the Western Pacific, Manila, Philippines; 6 Department of Immunizations, Vaccines and Biologicals, World Health Organization, Geneva, Switzerland; 7 PATH, Seattle, WA USA; 8 National Institute for Health Research Health Protection Research Unit in Emerging and Zoonotic Infections, and Institute of Infection and Global Health, University of Liverpool, Liverpool, UK; 9 Walton Centre NHS Foundation Trust, Liverpool, UK; 10 Armed Forces Research Institute of Medical Science, Bangkok, Thailand; 11 Centers for Disease Control and Prevention, Atlanta, GA USA; 12 Bridges to Development, Vashon, WA USA; 13 World Health Organization – South-East Asia Regional Office, New Delhi, India; 14 International Vaccine Institute, Seoul, South Korea; 15 Ministry of Public Health, Bangkok, Thailand

**Keywords:** Infectious diseases, Public health

## Abstract

Vaccines against Japanese encephalitis (JE) have been available for decades. Currently, most JE-endemic countries have vaccination programs for their at-risk populations. Even so, JE remains the leading recognized cause of viral encephalitis in Asia. In 2018, the U.S. Centers for Disease Control and Prevention and PATH co-convened a group of independent experts to review JE prevention and control successes, identify remaining scientific and operational issues that need to be addressed, discuss opportunities to further strengthen JE vaccination programs, and identify strategies and solutions to ensure sustainability of JE control during the next decade. This paper summarizes the key discussion points and recommendations to sustain and expand JE control.

## Introduction

Despite the availability of safe and effective vaccines against Japanese encephalitis (JE) for several decades (Table 1), JE virus (JEV) remains the leading recognized cause of viral encephalitis in Asia^1^. JEV is transmitted by mosquitoes and is sustained in an enzootic cycle with pigs and wading birds as amplifying hosts. Human cases typically occur in children and in rural areas where people live and work in proximity to elements in the transmission cycle, such as near rice fields or domestic pigs. There is a risk of JEV transmission in 24 countries in Asia and the Western Pacific, either nationally or in endemic areas^2^. While some regions have nearly eliminated JE through comprehensive immunization programs, the incidence in other areas remains high^3^.

**Table 1:** Licensed Japanese encephalitis (JE) vaccines.

Vaccine type	Substrate	Viral strains	WHO-prequalified manufacturers	WHO recommended vaccine schedule	Other manufacturers (domestic and traveler markets)
Inactivated	Mouse brain	Nakayama	n/a	n/a	Vietnam: Vabiotech
	Vero	Beijing-1	n/a	n/a	Japan: Biken (Research Foundation for Microbial Diseases of Osaka University), Kaketsuken (The Chemo-Sero-Therapeutic Research Institute, Korea: Boryung Pharmaceutical Co., Ltd (fill-finish of Kaketsuken vaccine)
	Vero	Beijing P-3	n/a	n/a	China: Liaoning Chengda Biotechnology Co
	Vero	SA 14-14-2	India: Biological E	Two doses 28 days apart, from 1 year of age	Austria: Valneva Austria GmbH
	Vero	Kolar strain (JEV 821564XY)	n/a	n/a	India: Bharat Biotech
Live attenuated	Hamster kidney cells	SA 14-14-2	China: Chengdu Institute of Biological Products	Single dose at ≥8 months of age	Wuhan Institute of Biological Products
Live chimeric	Vero	JE SA 14-14-2/yellow fever 17D	Thailand: Government Pharmaceutical Organization-Merieux Biological Products Co.	Single dose at ≥9 months of age^a^	n/a

Adapted from Halstead et al.^1^.

^a^Manufacturer recommends pediatric booster 1–2 years after the primary dose.

While most human JEV infections are asymptomatic, severe disease occurs in about 1 per 250 JEV infections^1^. JEV infection can rapidly progress to encephalitis, with an estimated case fatality rate of 20–30%^4,5^. Common symptoms include sudden onset of high fever, chills, headache, myalgias, mental confusion, and convulsions in pediatric patients^6^. Among survivors, about 30–50% have long-term neurologic sequelae with intellectual or physical disabilities^7,8^. Although there is no specific treatment for JE, supportive care improves outcome. JE burden estimates suffer from poor surveillance resulting from the difficulty of diagnosing JE without cerebrospinal fluid (CSF) samples and because cases generally occur in rural areas where surveillance and laboratory capacity might be limited.

The World Health Organization (WHO) has long recommended JE vaccination programs in areas where JE is a public health problem^9^. However, poor recognition of the burden of disease, prioritization of other vaccines and public health interventions over JE vaccination, and the high cost and multiple-dose regimen of the older, inactivated mouse brain-derived vaccine limited JE vaccine introduction. Since 2003, PATH and multiple stakeholder organizations have worked to expand the use of WHO-prequalified JE vaccines primarily through the support of Gavi, the Vaccine Alliance^10^. Substantial impact on JE-associated deaths and disability has been shown in countries administering JE vaccine in childhood vaccination schedules. For example, large vaccination campaigns targeting variable age ranges were implemented in 31 districts in Nepal from 2006 through 2011. JE incidence after the campaigns was 78% lower than the pre-campaign incidence, resulting in over 3000 JE cases estimated to have been prevented through 2014^11,12^.

In 2018, the U.S. Centers for Disease Control and Prevention and PATH co-convened a group of independent experts to review JE prevention and control successes, identify remaining scientific and operational issues that need to be addressed, discuss opportunities to further strengthen JE vaccination programs, and identify strategies and solutions to ensure sustainability of JE control during the next decade. This paper summarizes the key discussion points and recommendations to sustain and expand JE control (Box 1).

Box 1. Key recommendations for optimal JE control beyond 2020 *Reliable JE Vaccine Supply*

- Optimize and ensure a reliable global supply of affordable JE vaccines
  *Global Coordination and Policy Optimization*
- Establish and support an international coalition of partners with responsibility for global coordination of JE control
- Maintain dedicated opportunities for information exchange and discussion of emerging issues across country JE programs
- Support advocacy efforts to ensure momentum for JE control is not lost
  *JE Surveillance, Vaccine Utilization, and Diagnostics*
- Provide technical support to countries for JE surveillance and vaccine monitoring (e.g., coverage surveys, immunization registries)
- Support country efforts for surveillance and vaccine introduction, and quality assurance for laboratory testing
- Monitor JEV geographic spread as land use and climate change might affect mosquito distribution
  *Other Country Programmatic Support*
- Troubleshoot vaccine supply issues
- When possible, integrate surveillance and vaccination programs into other programs for efficiency and sustainability
- Conduct community advocacy and communication for maximal vaccine acceptance
- Provide guidance for the adverse event following immunization investigations of special interest for JE
  *Research Priorities*
- Monitor duration of protection following a single dose of live JE vaccines
- Assess vaccine safety in pregnant and immunocompromised persons vaccinated with live JE vaccines
- Standardize and validate plaque reduction neutralization test reagents and procedures
- Ensure reliable supply of JE diagnostic kits
- Facilitate the development of JE treatments
- Estimate JE vaccine impact globally to highlight the success of JE vaccine programs and the need to sustain progress made
- Study safety and immunogenicity of JE vaccine co-administration with any new vaccines that might be co-administered with JE vaccine

## Achievements in JE vaccine development, prequalification, and policy advancement

The first inactivated mouse brain-derived vaccines were developed in the 1930s. Vaccine manufacturers in several Asian countries developed these vaccines primarily for domestic use. Studies of early JE vaccines have shown that human illnesses can be eliminated with high vaccination coverage even though JEV continues to circulate in the enzootic cycle. For example, in Japan, universal vaccination with inactivated mouse brain-derived vaccine virtually eliminated JE in the early 1990s^12^.

In the 1980s, virologists in China developed SA 14-14-2 (CD-JEV), a live attenuated JE vaccine grown on primary hamster kidney cells. This vaccine was introduced province by province and markedly reduced JE incidence in China^13^. Recognizing the potential impact with greater use of CD-JEV, in 2003, PATH partnered with the Chengdu Institute of Biological Products (CDIBP) to export CD-JEV internationally. CDIBP and PATH developed a new manufacturing facility compliant with good manufacturing practices (GMP) and greater production capacity, which opened in 2012. As part of this partnership, CDIBP also made CD-JEV available to low- and lower middle-income countries at a low public sector price to facilitate countries’ abilities to fund JE vaccine introduction.

Defining an immune correlate of protection (i.e., neutralizing antibody titer ≥1:10) in 2005 was a critical step in JE vaccine development^14^. In a landmark vaccine efficacy study of mouse brain-derived JE vaccine conducted in Thailand, more than 65,000 children were vaccinated^15^. Due to the high cost of clinical trials, any subsequent efficacy trial would be expensive and likely infeasible due to the need to vaccinate and clinically follow so many children. However, defining an immune correlate of protection allowed manufacturers to develop new JE vaccines for commercial use without large and logistically challenging vaccine efficacy trials. Several Asian and European manufacturers developed inactivated Vero cell culture-based JE vaccines that had a superior safety profile and were easier to manufacture than mouse brain vaccine, but, like earlier inactivated vaccines, still required multiple doses and periodic boosting to maintain immunity. Another manufacturer developed a recombinant chimeric vaccine (JE-CV) by inserting the E and prM genes of SA 14-14-2 JEV into a yellow fever 17D vaccine backbone^1^. Thus, in a few decades, the JE vaccine portfolio expanded to multiple products using a variety of vaccine technologies.

WHO prequalification is a process to validate a product’s quality, safety, and efficacy, and to ensure a manufacturer is compliant with WHO and international quality standards^16^. Prequalification is needed for vaccines procured through UNICEF, procured using Gavi support, or intended for use in WHO and other United Nations programs. Biological E. Ltd’s JEEV, a Vero cell culture-based vaccine, was prequalified in 2013, but initially was indicated for adults only. Later in 2013, CD-JEV became the first JE vaccine prequalified for children and the first Chinese vaccine to be WHO prequalified, an accomplishment that may help additional Chinese-manufactured vaccines enter the global markets.

**Fig. 1:**
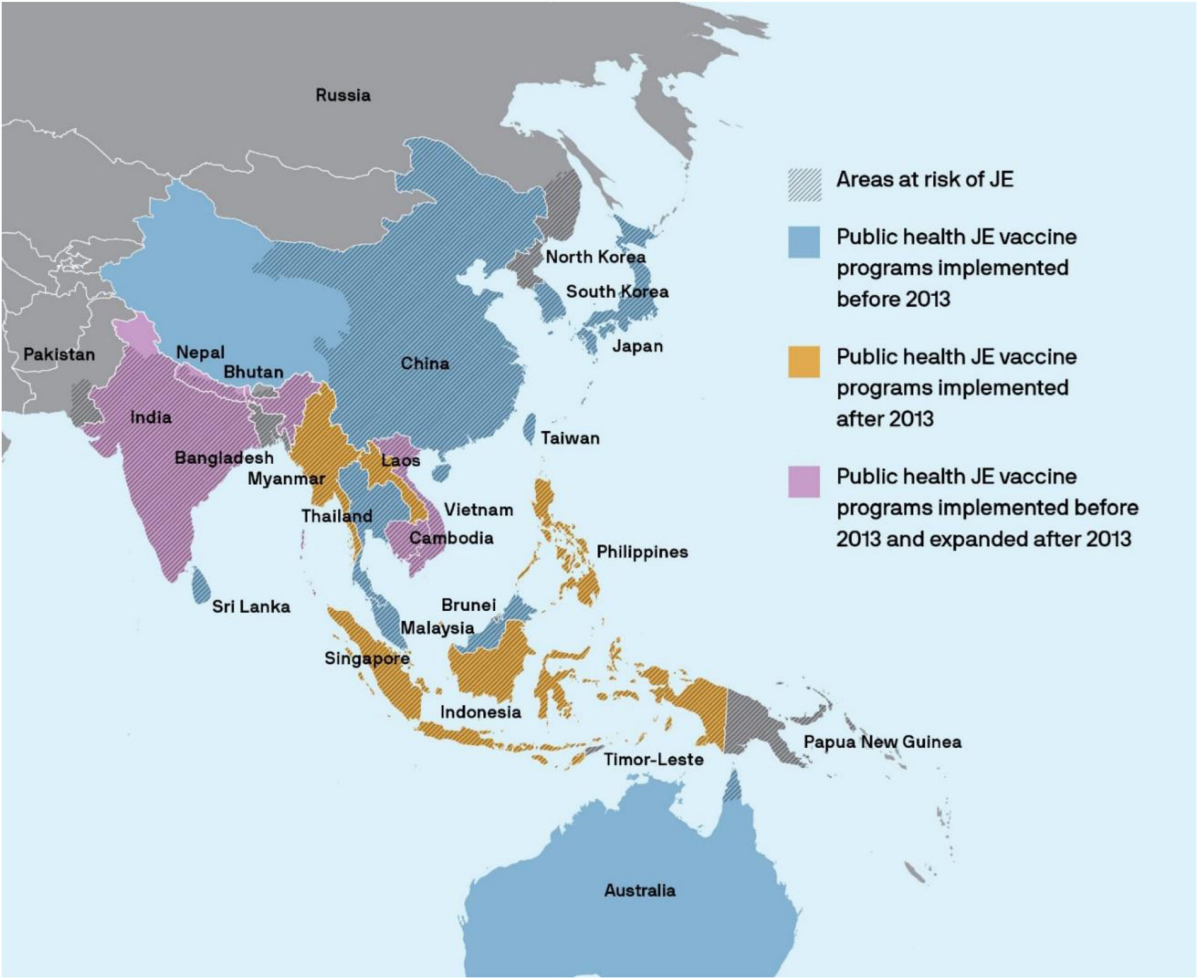
Areas with risk of Japanese encephalitis (JE) transmission and JE vaccine introduction relative to the WHO JE vaccine prequalification year (2013). While some countries have implemented national public health JE vaccine programs, others may have only introduced in high risk areas.

After WHO prequalification of these JE vaccines, Gavi committed to financing catch-up vaccination campaigns followed by routine vaccination within the childhood vaccination schedule, allowing Gavi-eligible countries to introduce JE vaccine. New vaccine introductions and expansions of JE vaccination programs were also enabled by the WHO recommendation that JE vaccine be introduced in all areas where JE is a public health priority, improved JE awareness at country and community level, government commitment, and financial and/or technical support by organizations such as WHO, PATH, Gavi, and the Bill and Melinda Gates Foundation^3^. Guidance and training materials were developed to support country decision-making and vaccine introduction as well as JE surveillance and measurement of vaccine impact^17–19^. With products available and funding mechanisms in place, accelerated JE control became one of eight immunization goals in the Regional Framework for Implementation of the Global Vaccine Action Plan in the Western Pacific, highlighting JE as a regional priority and defining operational targets for accelerated control of JE to be achieved by 2030^20^. As of June 2020, 15 countries have national or subnational public health vaccination programs in JE-endemic areas. These include Australia (Outer Torres Strait Islands), Malaysia (Sarawak), Japan, Republic of Korea, Thailand, Cambodia, Lao PDR, Myanmar, Indonesia (Bali), Philippines (three high-incidence regions), China, India (about 40–50% of districts), Nepal, Sri Lanka, and Vietnam (Fig. 1).

## Status of JE surveillance and control

JEV is a positive-stranded, enveloped RNA flavivirus. Because of low virus titers in infected humans and the neurotropism of JEV, the virus is rarely isolated from human cases. Therefore the gold standard for JE diagnosis is the presence of JE immunoglobulin M (IgM) antibody in the CSF^1^. However, as a disease that occurs predominantly in rural areas, the capacity for CSF collection, especially in children, is often limited and lumbar punctures are not always performed, especially in settings where treatment for encephalitis is limited or not available.

Access to diagnostics is important because JE cannot be clinically differentiated from other viral causes of acute central nervous system infection such as enterovirus 71^21^. Important progress in improving JE surveillance and defining disease burden came with the development of a range of JE diagnostic kits^22–25^. While three commercial JE IgM ELISA assays were previously available in JE-endemic countries, only the InBios JE Detect^TM^ IgM antibody capture ELISA is still being manufactured. CSF can be tested with the InBios JE assay alone, but, because of cross reactivity, serum samples must also be tested with a dengue IgM assay and results interpreted according to a CDC-devised algorithm^26^.

Even when diagnostics are available, interpreting serum IgM results in areas with co-circulation of other flaviviruses can be complicated because antibodies against other flaviviruses often cross react with JEV. In addition, it is well-recognized that IgM following JEV infection can be detectable for months or years, so there is sometimes uncertainty when JE IgM is detected in serum whether it truly reflects JEV as the cause of a patient’s presentation or could be persisting IgM following a previous asymptomatic infection or mild febrile illness unrecognized as JE. A recent analysis of IgM following vaccination with CD-JEV in a clinical trial suggests serum IgM is common at 28 days following vaccination but rare at 6 months following vaccination (~40% vs. 3% of vaccinees with positive or equivocal test results by ELISA, respectively)^27^. While this finding suggests that serum IgM is unlikely to be related to vaccination administered at least six months prior, it does not negate the importance of CSF collection for proper JE diagnosis.

To improve and standardize JE diagnosis in JE-endemic countries, PATH worked with partners to establish WHO JE Laboratory Networks in WHO’s South-East Asia and Western Pacific Regions during 2006–2008. These networks have substantially increased the frequency and quality of JE laboratory testing and continue to provide support to countries for confirmatory testing, quality assurance, and other JE testing issues^28^.

Despite progress with JE diagnostics and laboratory testing, because of the difficulty diagnosing JE, reported JE case counts are generally considered to be underestimates of true JE incidence. A 2011 estimate of global JE burden, in which high-quality surveillance data from limited areas in Asia were extrapolated to other endemic regions, calculated that 67,900 JE cases occurred annually, of which 75% occurred in children <15 years of age^29^. Based on the reported case fatality rate of 20–30% and long-term neurological sequelae among 30–50% of survivors, it was estimated that JE caused 13,600–20,400 fatalities each year and long-term neuropsychological sequelae in 14,300–27,200 people. In contrast to the estimate of case numbers, only 4402 JE cases were reported through the WHO-UNICEF Joint Reporting Form on Immunization in 2018, suggesting substantial under-reporting^2^. In a vaccine impact assessment conducted in Nepal using JE and acute encephalitis syndrome (AES) surveillance data, AES incidence was 59% lower than expected had no JE vaccination campaign occurred, with the absolute reduction in AES cases substantially greater than the reduction in confirmed JE cases, suggesting many AES cases were undiagnosed JE cases and again highlighting the unrecognized burden of JE^11^.

In addition to having better burden of disease estimates, JE surveillance is critical to inform vaccine introduction, monitor vaccination programs and identify alternative causes of encephalitis, evaluate vaccine effectiveness in different epidemiological settings, and monitor JEV expansion into new areas. WHO first published JE surveillance standards in 2008, and they were updated in 2018^30,31^. Still, there continues to be substantial variability in approach, quality, and extent of surveillance in JE-endemic countries, based on local capacity and resources and the need for integration into pre-existing systems^2^.

JE has been considered a disease of children in JE-endemic countries; immunity to JE by natural infection is widespread by adulthood and generally believed to be lifelong, at least in the context of natural boosting in endemic areas^32^. While vaccination has contributed to a relative shift in cases to older age groups in some countries, other countries without vaccination programs have also shown a substantial proportion of cases in adults, raising the question of the need for adult vaccination^32,33^. JE vaccination programs in Nepal that targeted all age groups showed the rate of AES in individuals ≥15 years after vaccination was 77% lower than expected, suggesting an unrecognized burden of JE in this age group. While strong pediatric vaccination programs are the foundation of a JE vaccination program, some countries, based on the burden of disease and prioritization, may consider vaccinating adults as well.

Mosquito-borne transmission is of greatest concern, although transplacental transmission and transmission through blood transfusion have been documented^34,35^. As a mosquito-borne disease, JE incidence is highly variable based on temperature, elevation, and rainfall. JEV is transmitted primarily by the mosquito *Culex tritaeniorhynchus*. There have been limited reports of JEV outside of Asia. In Italy, one pool of *Culex pipiens* was found to contain JEV as determined by RT-PCR of a small JEV RNA fragment, although no virus isolation was performed. In 2016, a patient with confirmed yellow fever infection in Angola was found to be co-infected with a JEV genotype III virus based on deep sequencing technology^36,37^. Further data are needed to confirm JEV transmission outside of Asia; however, with the global presence of Culex vectors and amplifying hosts, it is possible JEV could emerge outside of the traditional geographic boundaries due to population movement into nonendemic areas that results in changes in local farming practices and animal husbandry, expanded rice irrigation to meet population demand, and possibly global climate change. Although less likely, the transport of infected mosquito vectors or their infected eggs, the importation of viremic vertebrate hosts, or changes in bird migration patterns due to global climate change has the potential to expand the range of JEV^38,39^.

## Status of JE vaccines and vaccination

There have been ~15 JE vaccine products commercialized in Asia, including several manufactured for use only in the country of manufacture^1,40^. JE vaccines fall into four categories: (1) live attenuated vaccine (i.e., CD-JEV); (2) live recombinant (chimeric) vaccine (i.e., JE-CV); (3) inactivated Vero cell-derived vaccine; and (4) inactivated mouse brain-derived vaccine. WHO recommends both CD-JEV and JE-CV be administered on a single-dose schedule starting at age 8 months and 9 months, respectively, although the JE-CV vaccine package insert recommends a pediatric booster dose. WHO recommends administering Vero cell JE vaccines per the package insert, which is generally 2–3 doses with a booster dose at variable time intervals by product. CD-JEV holds the greatest global market share with more than 300 million doses to date administered outside of China. WHO recommends countries move away from mouse brain vaccines given the favorable profile of second-generation products, and no mouse brain vaccines are WHO prequalified^3^.

PATH chose CDIBP’s CD-JEV to target for global use because it could be administered as a single-dose vaccine and marketed at an affordable price in low-income countries in Asia. Data from China supported high vaccine effectiveness with a strong safety profile and PATH led additional clinical trials in other Asian countries in accordance with international standards, as did other manufacturers using CD-JEV as a comparison vaccine^41–44^. Studies using CD-JEV vaccine generally showed seroprotection (i.e., neutralizing antibody titer ≥1:10) in more than 90% of vaccinees 28 days after primary vaccination, although one study using WHO-prequalified CD-JEV found lower seroprotection rates^43–48^. Most case-control studies of CD-JEV vaccine demonstrated vaccine effectiveness at around 95% up to five years post-vaccination, although other studies have found lower point estimates^49–56^. Poor ascertainment of JE cases and accurate vaccination status is a challenge for these studies. A vaccine effectiveness study of the WHO-prequalified CD-JEV vaccine is currently underway. Safety of CD-JEV has been assessed both in clinical trials and through post-licensure passive reporting of adverse events and the vaccine has been found to have a robust safety profile^57^. Reports of encephalitis post-vaccination with CD-JEV are rare and a causal relationship with vaccination has not been established^58,59^. The WHO Global Advisory Committee on Vaccine Safety reviewed data on CD-JEV and determined it has a strong safety profile based on available data^60^.

Countries use different vaccines in country-supported JE immunization programs (implemented either nationally or regionally): 11 countries use CD-JEV (Cambodia, China, India, Indonesia (Bali), Laos, Myanmar, Nepal, Philippines, South Korea, Sri Lanka, Thailand), four use JE-CV vaccine (Australia, Malaysia, Thailand, and China’s Taiwan province), and three use Vero cell vaccines (Japan, South Korea, and China’s Taiwan province). Vietnam continues to use mouse brain vaccines. Brunei and North Korea have used JE-CV and CD-JEV, respectively, but do not have sustained programs, and Singapore has determined routine vaccination is not required based on surveillance data. Bangladesh, Bhutan, Pakistan, Papua New Guinea, Russia, and Timor Leste do not have JE immunization programs, but have recent or past evidence of JEV transmission^61^. Several countries co-administer CD-JEV vaccine with the measles-containing vaccines, which has been shown convincingly not to cause immune interference^46,47,62^.

## Programmatic challenges and threats to JE vaccination

Despite great advances in JE vaccination and control over the last 15 years, there continue to be challenges that put these gains at risk and may impede further progress. These challenges fall into the categories of (1) maintaining high JE vaccine coverage, (2) maintaining a stable JE vaccine supply, and (3) maintaining surveillance and vaccination data.

### Maintaining high JE vaccine coverage

Because of the enzootic cycle of JEV involving birds and mosquitoes, JEV elimination from the environment is impossible. Sustained high vaccination coverage must be maintained to prevent human cases. There is also evidence JEV is moving into new geographic areas, including evidence of occasional urban transmission; consequently target areas where vaccination is needed may shift and grow^63–66^. Careful diagnoses and surveillance are needed to monitor JE geographic spread as human migration, land use and climate change may affect mosquito distribution in the future. Furthermore, general vaccine hesitancy among parents and healthcare providers is on the rise, including in countries affected by JE^67–69^. In 2019, WHO named vaccine hesitancy as one of the top 10 threats to global health^70^. Confidence in vaccination programs is essential to maintain high JE vaccine coverage.

### Maintaining a stable JE vaccine supply

CDIBP’s CD-JEV is the most widely used JE vaccine and several low- and middle-income countries rely on it. This reliance on a single product from one site operated by a lone manufacturer presents numerous threats to vaccine supply. Catastrophic events, such as infection among the pathogen-free colony of hamsters that are a source of cells for vaccine virus growth, natural disasters (e.g., earthquakes), or human disease outbreaks (e.g., pandemic influenza) could threaten the global vaccine supply. A vaccine supply continuity plan to help ensure CD-JEV global supply and availability is needed, using methods such as off-site bulk storage and identification of alternative sources of manufacturing materials.

There are issues of supply and demand due to the need for manufacturers to grow virus for either live or inactivated vaccines. Thus, countries must assess their need for vaccines many months in advance of usage and are unable to acquire WHO-prequalified JE vaccines in a rapid ad hoc fashion, which makes ordering vaccines as a response to a JE outbreak unfeasible. None of the three WHO-prequalified vaccine producers have stockpiles of finished products with which to respond to requests during outbreaks. For example, the time required to produce and release a batch of CD-JEV can be up to nine months; as a result, countries must order at least nine months in advance. Another challenge with CD-JEV is the short shelf life of 24 months (compared to 36 months for the other two WHO-prequalified products). This means CD-JEV must be used expeditiously once it arrives in countries.

WHO currently recommends that CD-JEV be given as a single-dose vaccine without need for a booster dose. India and China, large consumers of CD-JEV, administer the vaccine as a two-dose primary series for programmatic reasons, which has an impact on vaccine supply. Because CDIBP has established a public sector price for specific JE-endemic countries, CD-JEV is currently the most viable vaccine product for many low- and lower middle-income countries. The cost and/or multiple-dosing regimens of other JE vaccine products have made them less desirable in many settings. Nonetheless, diversity is needed in the global supply of JE vaccine.

### Poor surveillance and vaccination data

Other important challenges exist with respect to disease surveillance and vaccine monitoring. Poor surveillance for JE disease has made the burden difficult to quantify in many countries, hindering decisions about vaccine introduction. When JE vaccination has been introduced, poor surveillance has limited availability of data to demonstrate impact and support advocacy efforts to maintain vaccine programs. Lack of immunization registries makes determination of vaccination status difficult over the longer term; monitoring vaccine failures and determining vaccination status in vaccine effectiveness studies are both critical for studying the duration of vaccine protection and the assessment of the eventual need of a booster dose. Challenges with the laboratory component of surveillance include suboptimal coordination between laboratory and epidemiology staff in some countries, and general challenges such as sample quality on arrival in laboratories, and difficulties with sample transport internationally for testing at reference laboratories. The risk of an insufficient supply of commercial JE IgM kits because of the limited market is a large threat to routine JE diagnostics. There remains no ready alternative if the only company currently producing a commercial IgM ELISA discontinues production. Critically, any reduction in support to countries from the regional JE laboratory networks could threaten the gains made in capacity for, and quality of, JE diagnosis.

Vaccine safety concerns, especially around severe adverse events such as encephalitis, can trigger vaccine hesitancy. This was seen in India when a cluster of suspected encephalitis cases were reported shortly after a JE vaccination campaign in 2014, though on review the cases were determined to be unrelated to vaccination^71^. It is important to have robust investigations of adverse events following immunization (AEFI) without creating unsupported concerns. Investigation of post-vaccination encephalitis has not been systematic and requires good epidemiologic and laboratory resources to investigate properly. Enhanced AEFI surveillance following the initiation of a JE vaccine campaign is important. Working with stakeholders to maximize communication efforts while at the same time minimizing misinformation in the community is critical.

Decisions about vaccine introduction in resource-limited settings frequently require prioritization of multiple public health interventions for diseases that affect populations. Without good JE burden data, it has been difficult to contextualize the importance of JE vaccination relative to other high-priority vaccines competing for government resources, such as rotavirus and human papillomavirus vaccines. Continued advocacy is necessary for keeping JE on the list of priority vaccines for countries to consider.

## Research gaps

### Vaccine immunogenicity and need for booster doses

Much of the earlier research on CD-JEV effectiveness and immunogenicity was done using vaccines produced in an older CDIBP facility that did not meet good manufacturing practice (GMP) standards. To license and prequalify CD-JEV made in the newer, GMP-compliant facility, a non-inferiority study compared three lots of “new facility” vaccine with one lot of vaccine from the older facility^48^. Seroprotection rates 28 days after vaccination for recipients of the new facility vaccine were 80.2–84.5% compared to 86.3% among recipients of the vaccine made in the older facility. Although this was not statistically different, seroprotection rates were noted to be higher (i.e., 90.5 to 92.1%) in earlier clinical studies of CD-JEV^47,72^. Because plaque reduction neutralization tests in these studies were performed at different times, comparing results is difficult. Still, while the findings of the lot-to-lot consistency trial supported the use of “newer facility” CD-JEV, continued monitoring is required on the vaccine’s duration of protection, which potentially could have implications for booster doses.

With reasonable immunogenicity of CD-JEV over a sustained period of time and an absence of reported vaccine failures with programmatic use, WHO does not recommend JE vaccine booster doses unless future evidence warrants it^40^. Data on vaccine effectiveness studies and vaccine failure monitoring are important to inform the need for booster doses for all WHO-prequalified JE vaccines. Studies are in progress (e.g., a case-control study in Bali, Indonesia) that will provide longer-term vaccine effectiveness data (e.g., up to 5 years) and inform the duration of protection of a single dose of CD-JEV. Studies of antibody persistence alone are insufficient as undetectable neutralizing antibody might not indicate the absence of protective immunity. For example, data from a trial of JE-CV indicated that at two years post-primary vaccination some children who did not seroconvert with a primary JE-CV dose exhibited an anamnestic response following a receipt of a booster dose^40^. Similar findings were seen in a study of the other live JE vaccine, CD-JEV. Children who did not seroconvert initially or no longer had seroprotective antibody four years after immunization were given a booster dose of CD-JEV that resulted in a strong anamnestic response^73^. Anamnestic responses following revaccination with live JE vaccines suggest that despite undetectable antibodies, vaccines might be protected from clinical disease. Should booster doses of CD-JEV ultimately be recommended, this would have additional implications for vaccine supply and programmatic implementation. The anamnestic response has not been studied for inactivated JE vaccines.

Furthermore, data on safety and immunogenicity following vaccination of special populations, such as pregnant women and immunocompromised persons, are missing for both live vaccines, CD-JEV and JE-CV. Research priorities across vaccine platforms are available in WHO’s position paper on JE vaccines^3^.

### Emerging genotypes

All licensed vaccine viruses are genotype III JEV. While studies suggest these vaccines provide good protection against multiple genotypes, there are questions pertaining to future vaccine effectiveness if a new JEV genotype emerges^74,75^. Two genotype V JEV isolates have been identified, one from a human and one from a mosquito, and genotype V sequences from *Culex* mosquitoes also have been recently identified^76^. Additional evidence is required to determine if this genotype is emerging. If so, the question has arisen if currently available vaccines might be less effective, based on neutralizing antibody data and the 9% amino acid difference between genotype V and genotype III viruses. This situation should be monitored.

### Disease sequelae and treatment options

Although the expert meeting focused on JE vaccination, there are other research gaps related to JE and treatment. While disability following encephalitis has long been recognized, the extent of sequelae following mild, non-neurologic JE illness is poorly understood. Such data could provide a better understanding of the full burden of JE morbidity and a more comprehensive understanding of the benefits of vaccination. Better information on the comparative severity of JE in adults and children would also be useful. Finally, as there is no specific treatment for JE currently available, investigation of possible JE treatments is another important avenue for further research. Limited clinical trials to date have not yielded any successful compounds, although promising targets exist^77^. There is a potential role of inflammation as a contributor to poor clinical outcomes from JEV infections, thus anti-inflammatory agents in combination with antiviral agents might be effective; this deserves further study^77^.

## Recommendations to optimize JE control

The challenges defined above highlight that despite major achievements in JE control, efforts are required to continue and sustain progress. Experts at the meeting identified key recommendations in the following categories: (1) vaccine supply, (2) global coordination and policy optimization, (3) JE surveillance and diagnostics, (4) country programmatic support, and (5) research priorities.

### Reliable JE vaccine supply

An adequate supply of JE vaccine is essential to JE control and is currently at risk as discussed above. A reliable JE vaccine supply requires vaccination schedules be optimized, countries improve lead-time for ordering JE vaccine, and vaccine manufacturers develop contingency plans for events such as natural disasters or other disruptions of vaccine supply. JE vaccine manufacturers with pre-qualified products currently in limited use globally are encouraged to make their products available and affordable to affected countries. At a minimum, country licensure of more than one product will allow product switching if the need arises.

### Global coordination and policy optimization

A coalition of key partners should be established that is responsible for exchange of information, best practices, and coordinated actions among JE control programs. This coalition would serve as a technical resource for countries and should maintain a web-based library of materials to support vaccine introduction and monitoring. As national and international partners have highlighted the utility of biannual WHO JE biregional (i.e., South-East Asia and Western Pacific regions) meetings to share experiences and lessons learned, these meetings should continue under the coordination of the coalition. Biregional meetings are important for information exchange and discussion of new or emerging issues. Advocacy for JE vaccine introduction is still needed to ensure that the momentum achieved through the efforts of PATH, WHO and its regional offices, and other partners, WHO prequalification of JE vaccines, and Gavi support, is not lost. As additional data become available (see Research Priorities), re-evaluation of global JE vaccine policies (e.g., the need for booster doses) might be warranted.

### JE surveillance, vaccine utilization, and diagnostics

The need for strong surveillance and diagnostics does not end when a vaccine is introduced in the country. Many JE-endemic countries will still need support in the areas of JE surveillance, diagnostics, and vaccination program monitoring after JE vaccine has been introduced. JE surveillance support will help decision-making for countries planning to introduce JE vaccine to understand disease burden and target vaccination geographically and by age. In countries with JE vaccination, JE surveillance and immunization registries are important to help identify program deficiencies, assess long-term vaccine protection, and monitor the geographic spread of JE. JE vaccination program monitoring should be improved through vaccine coverage surveys and better completion of the WHO/UNICEF Joint Reporting Form on Immunization.

### Other country programmatic support

WHO Regional offices need to be supported as they directly assist countries with these issues—surveillance and vaccine introduction, quality assurance for laboratory testing, and troubleshooting vaccine supply issues. To maximize efficiencies across programs and ensure stable funding, efforts in surveillance, diagnostics, and vaccine coverage monitoring should be combined for JE and other vaccine-preventable diseases; this will also help integrate JE into routine immunization activities. Community advocacy and communication must be supported for maximal vaccine acceptance, especially during vaccination campaigns. To help address vaccine safety concerns and vaccine hesitancy, all reported serious AEFIs should be investigated promptly and properly, but with the care that the investigations do not create inappropriate concern that they are caused by vaccination if other etiologies are more likely. Readily available resources to guide AEFI investigations, particularly of encephalitis post-vaccination, would be helpful.

### High-priority research needs

- The potential need for a JE vaccine booster dose to achieve life-long protection against JE. Longer-term vaccine effectiveness studies and documentation of vaccine failures through careful JE surveillance will be the most convincing data to indicate if booster doses are required for CD-JEV or JE-CV and the frequency of boosting for inactivated JE vaccines.
- Vaccine safety in special populations. To address research gaps on vaccine safety in immunocompromised persons and pregnant women who are knowingly or unknowingly vaccinated with CD-JEV and JE-CV, vaccine registries for these populations are needed.
- Laboratory and diagnostic issues. Additional diagnostic tests are urgently needed. Market shaping for JE ELISA kits will likely be needed through Gavi and other agencies, as other manufacturers have not shown interest in the development of similar kits due to the limited market. The use of new technologies and platforms for diagnostics could hold promise in the future but would require investments. Standardized and validated plaque reduction neutralization test reagents and procedures may allow comparison of immunogenicity results across laboratories.
- JE treatment. To help facilitate JE treatment research, expert consultation is needed with the aim of identifying the next drugs that should be tested in humans as well as to discuss the design of therapeutic trials, including types of studies, suitable locations, and outcomes to be measured.
- JE vaccine impact. To document the importance of JE vaccination and the need for sustained efforts, it will be important to conduct a retrospective impact study of the number of JE cases, disabilities, and deaths averted and to estimate the total cost savings from JE vaccination. Mathematical modeling of expected JE cases in the absence of vaccination could be a potential approach to overcome the deficiencies in JE surveillance. Such studies could consolidate country support for existing JE immunization programs and place JE in proper context alongside other vaccine-preventable diseases to support decision-making for JE vaccine introduction.
- Safety and immunogenicity of JE vaccine co-administration with new vaccines. Ongoing safety and immunogenicity studies of co-administration of JE vaccines with newly introduced vaccines (e.g., typhoid conjugate vaccine) should be conducted when there is a programmatic desire to co-administer vaccines.

## Conclusions

JE control in Asia through immunization programs is a public health success. Together with affected country drive to develop new vaccines and control JE, international investments into an Asian domestic vaccine manufacturer enabled an affordable product and expanded access to this life-saving vaccine. However, more work is needed to secure the gains made and accelerate control of JE by expanding the use of JE vaccines in JE-endemic areas that do have immunization programs or do not have adequate programs.

## Data Availability

No datasets were generated or analyzed during the current study.
